# Ancestral mitochondrial N lineage from the Neolithic ‘green’ Sahara

**DOI:** 10.1038/s41598-019-39802-1

**Published:** 2019-03-05

**Authors:** Stefania Vai, Stefania Sarno, Martina Lari, Donata Luiselli, Giorgio Manzi, Marina Gallinaro, Safaa Mataich, Alexander Hübner, Alessandra Modi, Elena Pilli, Mary Anne Tafuri, David Caramelli, Savino di Lernia

**Affiliations:** 1 Department of Biology, University of Florence, Florence, Italy; 2 Department of Biological, Geological and Environmental Sciences, University of Bologna, Bologna, Italy; 3 Department of Cultural Heritage, University of Bologna, Ravenna, Italy; 4 Department of Environmental Biology, Sapienza University of Rome, Rome, Italy; 5 Department of Ancient World Studies, Sapienza University of Rome, Rome, Italy; 6 Max-Planck-Institute for Evolutionary Anthropology, Department Evolutionary Genetics, Leipzig, Germany; 7 School of Geography, Archaeology and Environmental Studies, University of the Witwatersrand, Johannesburg, South Africa

## Abstract

Because Africa’s climate hampers DNA preservation, knowledge of its genetic variability is mainly restricted to modern samples, even though population genetics dynamics and back-migrations from Eurasia may have modified haplotype frequencies, masking ancient genetic scenarios. Thanks to improved methodologies, ancient genetic data for the African continent are now increasingly available, starting to fill in the gap. Here we present newly obtained mitochondrial genomes from two ~7000-year-old individuals from Takarkori rockshelter, Libya, representing the earliest and first genetic data for the Sahara region. These individuals carry a novel mutation motif linked to the haplogroup N root. Our result demonstrates the presence of an ancestral lineage of the N haplogroup in the Holocene “Green Sahara”, associated to a Middle Pastoral (Neolithic) context.

## Introduction

Fossil remains attest to the presence of Anatomically Modern Humans (AMH) in the Near East around 130–100 kilo annum (ka), but their decisive spread from Africa all over the world seems to be the result of a migration that occurred after 70 ka^1–3^. African genetic variability is well known only for current times, but local population genetics dynamics and back-migrations from Eurasia could have modified haplotype frequencies, thereby masking ancient genetic scenarios^4–7^. Several variables, such as temperature, pH and salinity, influence molecular degradation^8,9^ and environmental conditions in hot and arid regions of Africa do not favour DNA preservation. For these reasons, only in the last years, thanks to methodological improvements in the ancient DNA field, genetic data from ancient human samples have been obtained for the African continent^10–16^.

**Figure 1:**
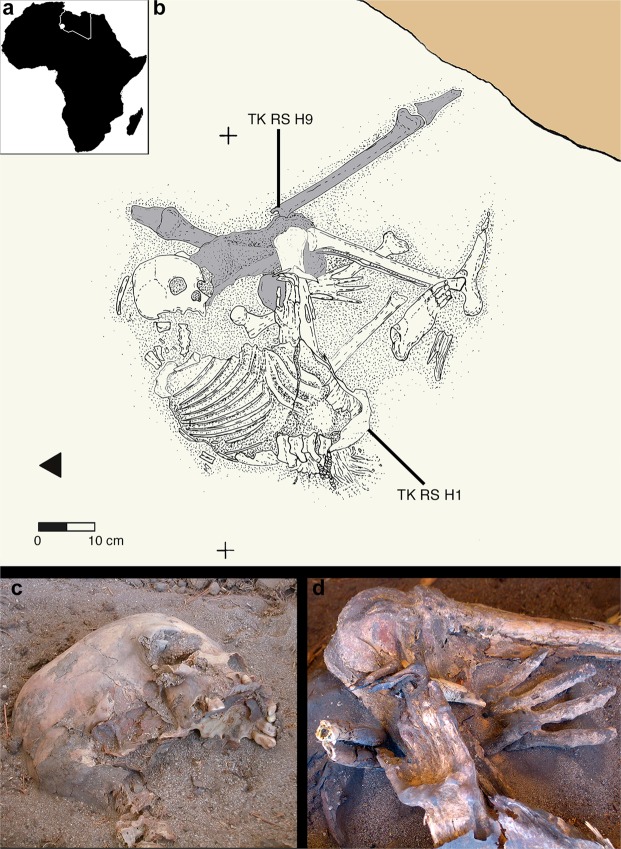
The ~7000-year-old individuals from Takarkori Rockshelter in their context. (**a**) Map of Africa with location of the site (white dot); (**b**) depositional relations of TK RS H1 and H9 (drawn by F. Del Fattore); details of skull (**c**) and hands (**d**) of TK RS H1.

Here we present the earliest and first genetic data from the Saharan region and contextualize them into the mitochondrial phylogeny, in order to enhance the understanding of past African genetic variability and human lineage evolution and dispersal. The analysed material comes from Takarkori rockshelter in the southern Tadrart Acacus massif of Libya’s central Sahara (Fig. S1). The rockshelter’s thick stratigraphy spans several millennia of the Holocene and documents the transition from hunting and gathering to pastoralism^17^. Fifteen burials mostly referred to the Early and Middle Pastoral (Neolithic) date to between ~8.9 and ~4.8 ka. Located in a recessed area of the shelter, these burials were exclusively of women of reproductive age, children and juveniles. Strontium isotope analysis revealed all have the same local geographic origin. These patterns suggest a kinship system based on matrilineal descent with repeated use of the rockshelter as deathplace by early pastoral communities^18^. The DNA analysis focuses on two individuals of Middle Pastoral age, who present signs of natural mummification: TK RS H1 and TK RS H9 (Fig. 1). The samples are directly radiocarbon dated to 6090 ± 60 BP (7.1–6.7 ka; 7159–6797 calBP, 95.4% probability) and 5600 ± 70 BP (6.5–6.2 ka; 6547–6280 calBP, 95.4% probability) respectively (Table S1). Both belong to adult females^18^. We extracted DNA from several bone and skin samples (Table S2) and performed a capture enrichment for the mitochondrial genome (mtDNA).

## Results and Dicussion

### Ancient DNA analysis

A tooth sample for TK RS H1 gave a mean coverage of the mtDNA of 72x with 99.99% of positions covered at least threefold (Table S3). Two bone fragments from the fibula of TK RS H9 gave a mean coverage of the mtDNA of 8x and 8.4x with 94.13% and 89.75% of positions covered at least threefold respectively (Table S3). Nucleotide misincorporation and fragmentation patterns of the molecules were consistent with the samples’ age and environmental conditions^8^ (Table S3, Figs S2, S3). Modern human DNA contamination is absent (Table S4).

### Phylogenetic analysis

**Figure 2:**
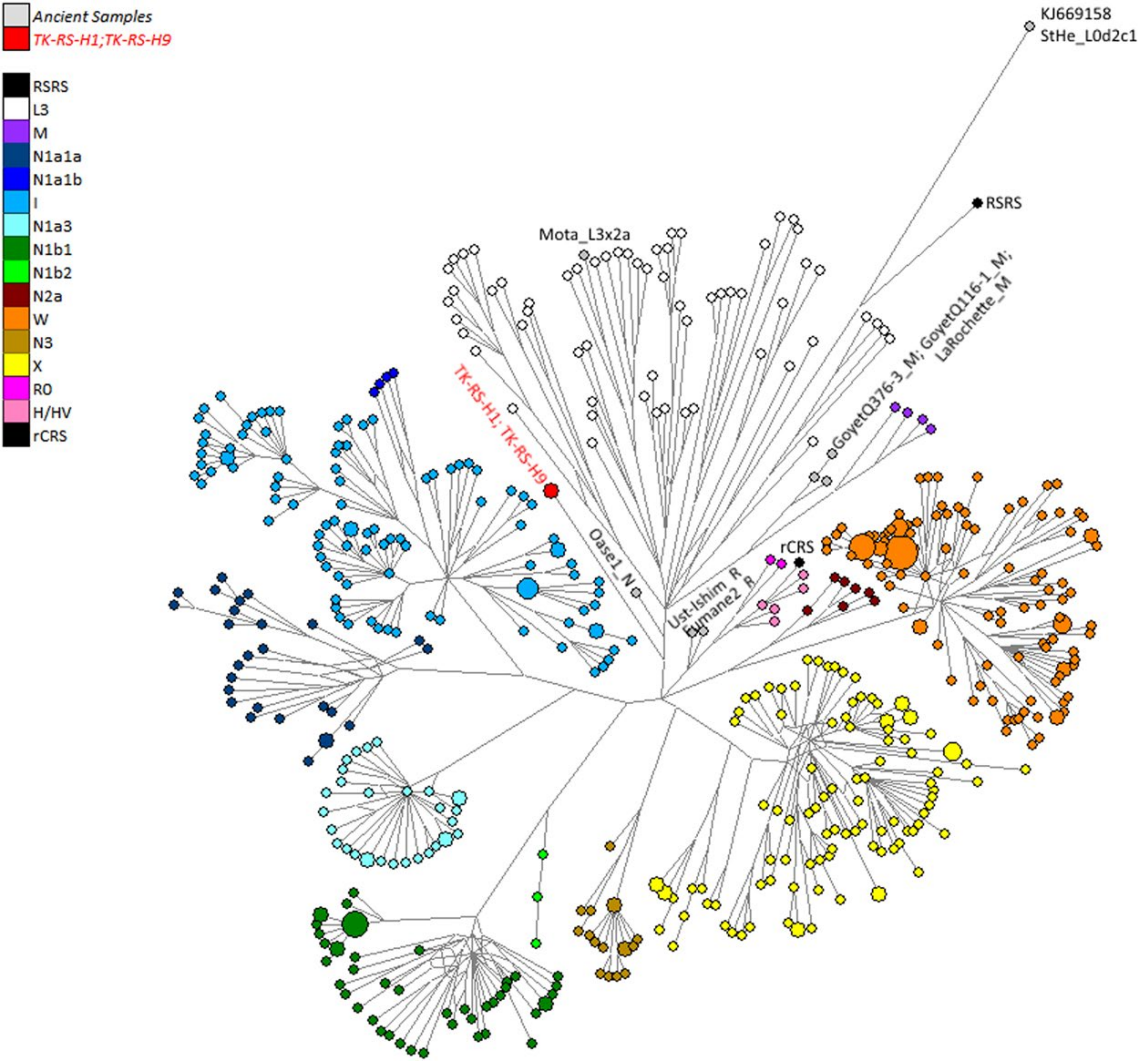
Median Joining Network representing phylogenetic relationships between the Takarkori samples TK RS H1 and TK RS H9 (in red), 8 ancient sequences (names are indicated at the nodes) and 538 modern sequences representing the major mitochondrial lineages and sub-lineages for N macro-haplogroup, indicated in different colours. The reference sequences rCRS and RSRS are represented with black nodes.

**Figure 3:**
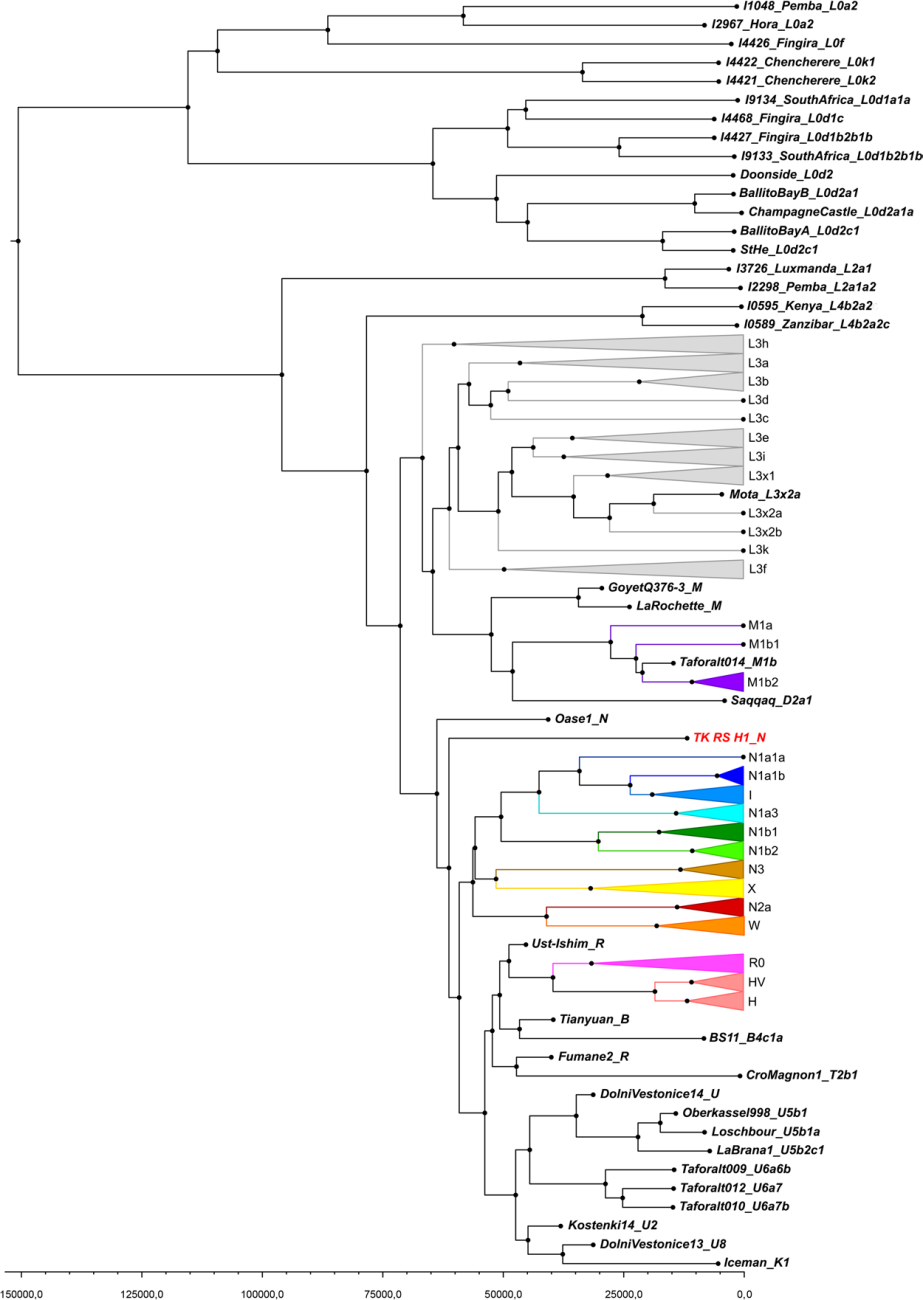
Phylogenetic tree constructed for the Takarkori sample TK RS H1 and 209 published complete genomes of ancient and modern samples. The major mitochondrial lineages and sub-lineages for N macro-haplogroup are indicated in different colours as in Fig. 2.

Comparing the consensus sequences of individuals TK RS H1 and TK RS H9, the same haplotype was found except for possible private mutations in TK RS H9 masked by missing data (Table S5). According to radiocarbon datings (Table S1) the two individuals were separated by a maximum of 879 and a minimum of 250 years, corresponding to 35–10 25-year-long generations, hence the perfect match of the sequences obtained sustains the hypothesis of close maternal kinship. This supports the cultural interpretation of the repeated use of the rockshelter to dispose of members of the same lineage^18^. The sequences were analysed regarding their position in the human mitochondrial phylogeny and were attributed to the N haplogroup with some private mutations. A Median Joining Network was drawn by combining the sequences obtained for TK RS H1 and TK RS H9 with an extended reference dataset of 536 modern individuals belonging to the different branches of macro-haplogroups N and L3, from which N originated. Further, we included 8 ancient European and African samples, selected among the published mitogenomes, with a special emphasis in basal informative haplogroups and added the rCRS and RSRS reference sequences for comparison (Fig. 2; Table S6). A Bayesian phylogenetic tree was inferred from a multi-sequence alignment of the mtDNA sequence of TK RS H1, as representative of the haplotype found in Takarkori, 167 modern samples with lineage relevance for the main haplogroups and sub-haplogroups and 42 published complete genomes of dated ancient samples as tip calibration points (Fig. 3; Fig. S4; Tables S6, S7). Both the Median Joining Network and the phylogenetic tree confirmed the attribution of the sample to the N haplogroup, highlighting it as a basal lineage, which branches off immediately after the Palaeolithic sample Oase 1^19^ and before all present-day N-derived mtDNAs. A mutation rate of 2.26 × 10^−8^ mutation/site/year (95% HPD: 1.85–2.68 × 10^−8^) was estimated by the Bayesian analysis, consistent with the results of previous studies^3,20^. The time to the most recent common ancestor (TMRCA) for N clade and for the branch leading to Takarkori sequence was estimated at 64,103 years BP (95% HPD: 54,535–74,578) and 61,618 years BP (95% HPD: 52,517–71,405) respectively. The tip date for the TK RS H1 terminal node was estimated to be 12,325 years BP (95% HPD: 1–25,119). The large confidence interval, presumptively affected by the small number of sites of the mtDNA genome and short evolutionary time scale, is consistent with the radiocarbon date of the sample taking into account the confidence intervals and provides further evidence for the authenticity of the sequence.

## Conclusions

Until now, the origin of African haplogroup L3, from which N originated, has been dated around 60–70 ka and its expansion in Eastern Africa linked with the exit of AMH from the continent. The M and N haplogroups, which lie at the base of Eurasian mtDNA diversity, are today globally distributed outside Africa and are dated to around 50–65 ka, very close to the ancestral L3 clade. Their divergence from it is commonly considered to have occurred outside Africa or during the expansion^1,2,21,22^. The Arabian Peninsula represents a possible area where this occurred and a cradle from which the new branches spread toward Eurasia and back to Africa, including N1a and R0a, both of which are found in East Africa^1,7,23,24^.

The uneven geographical distribution of existing data could bias the representation of real past genetic variability: sampling gaps characterize the African continent, and several studies focus only on particular haplogroups. Moreover, the past genetic scenario is still poorly known for this large and crucial area, and the ancient mitochondrial sequences available refers only to few sites distributed in Ethiopia, Egypt, Morocco, Kenya, Malawi, Tanzania and South Africa and do not extend back in time more than ~15000 years.

**Figure 4:**
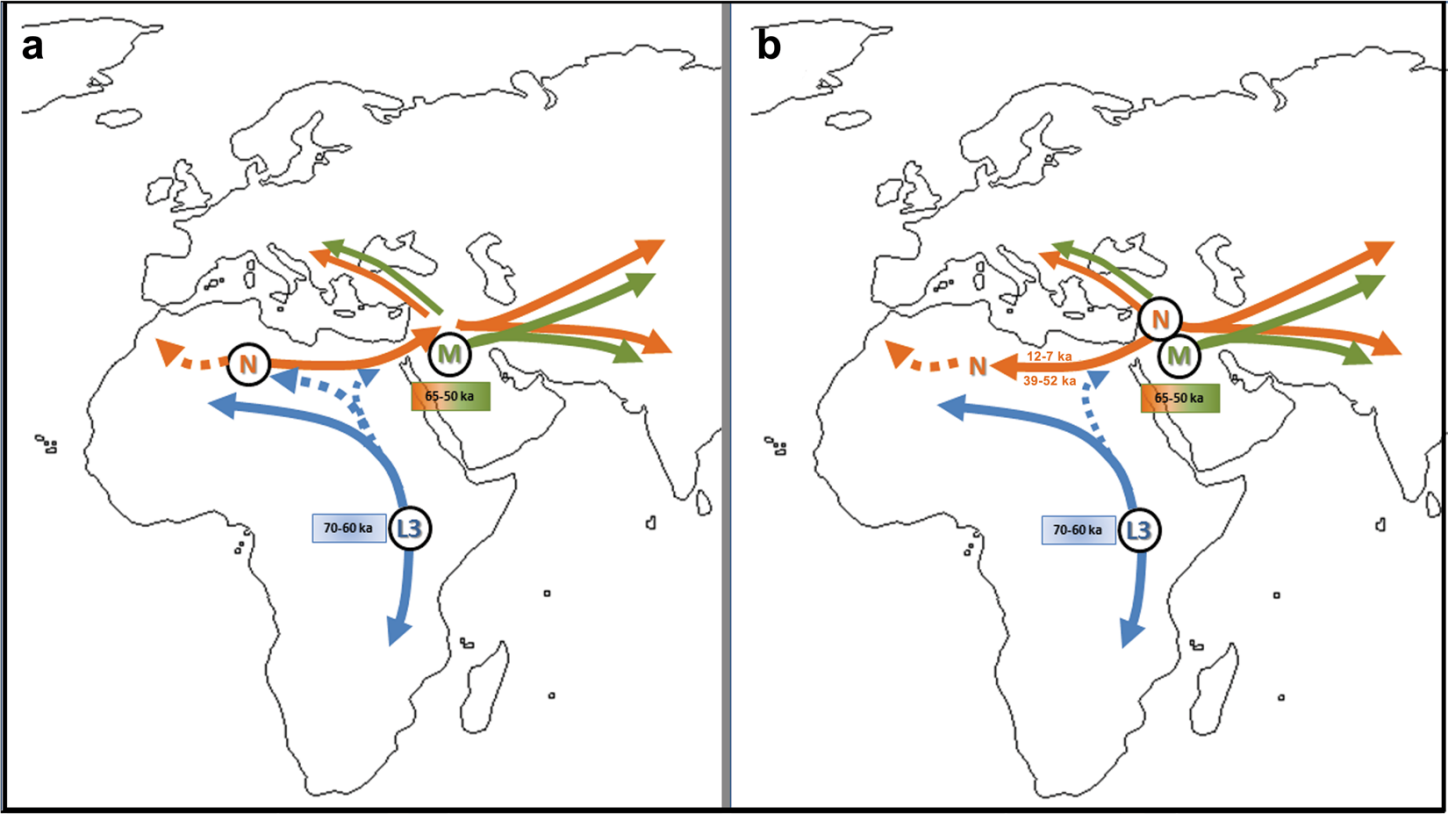
Map of Africa with the alternative models discussed. Haplogroups are indicated in black circles in their probable area of origin. Continuous arrows indicate spread by migration, while dashed arrows indicate molecular differentiation from one haplogroup to another. Dates of origin of haplogroups are indicated in squares. Dates along arrows indicate possible migration time. (**a**) Haplogroup N differentiates from L3 in the African continent, with a subsequent spread out of Africa. (**b**) Haplogroups M and N diverged from L3 outside Africa or during the expansion of AMH out of the continent; later migrations during Early Upper Paleolithic and the Neolithic diffusion led some lineages back to North Africa.

Our research reveals that the Neolithic Saharan individuals from Takarkori present a haplotype not previously identified in Africa, that belongs to a basal branch of haplogroup N. This discovery needs to be addressed cautiously, given its potential geographical, chronological and archaeological implications (Fig. 4). As recently suggested^23^, the presence of an unexpected branch where other clades prevail in the population may provide an indication of ancestry, but more data are necessary. The Saharan region was interested by strong climatic oscillations. Repeated peaks of humidity and the presence of several intermittent pulses of lake activity occurred between 125 and 11 ka^25^. Warmer and wetter environmental conditions characterized the Late Glacial Bølling/Allerød Interstadial^26^ allowing population growth and spread. The return of cooler, drier conditions during the Younger Dryas may have prompted human groups to exploit glacial refugia across this region. Interestingly the molecular date of the Takarkori sequence (12,325 BP) falls into the context of the Interstadial expansion. The analysed samples, dated to ~7000 BP, could represent a signal of a mitochondrial lineage that later disappeared because of genetic drift due to population contraction and isolation with the beginning of desertification^27,28^. A possible scenario envisages an introgression from Eurasia in ancient times that carried haplotypes that have since disappeared from Africa. The timing of this migration remains difficult to define. Late Pleistocene dispersal from Western Asia into Africa around 39–52 ka is suggested by the expansion of the U6 haplogroup^29,30^, with a potentially corresponding archaeological signature in the MSA Dabban industry of Cyrenaica, Libya, ca. 45–40 ka^31^. Individuals carrying a N haplogroup basal lineage could have followed the same dispersion pattern as U6: their legacy could have been survived up to ∼7000 years ago in the central Sahara thanks to the climatic conditions previously described, but replaced and disappeared in other parts of North Africa. Genomic data for seven 15,000-year-old individuals attributed to the Iberomaurusian culture in Taforalt (Morocco) suggest a connection with Epipaleolithic Natufians from Near East, while seem to exclude a possible gene flow from Upper Paleolithic Europe^32^. Our samples postdate the Taforalt individuals by up to 8,000 years and belong to Neolithic pastoral cultures of the Middle Holocene. It is known that livestock was introduced from Southwest Asia^33^ and early pastoralist connections between Northeast Africa and Arabia are indicated by a few sites along the Red Sea with sheep/goat dated to ~8.1–7.5 ka^34–36^. Thus, the spread of pastoralism from the Levant to Northeast Africa could probably represent the context for the introgression of the N haplogroup into the central Sahara, even if it is commonly associated with derivative lineages (N1)^1,37^. It is worth noting, however, that when geometric morphometric analysis of the skull of TK RS H1 is compared with a large published dataset it shows closer affinities with sub-Saharan contests^38^, such as Gobero in Niger whose occupation is dated from ~9.6–4.8 ka^39^. Unfortunately, no genetic data are available for this region that could help understanding the possible origin of the haplotype found at Takarkori.

Our results show an unprecedented evidence of an ancestral N lineage in the central Sahara, exactly at the middle of the north-south and east-west clines of the northern part of the continent, greatly enriching the fragmented picture of ancient DNA studies in Africa. The survival of an ancestral haplogroup in areas where it is now no longer present reinforces the need to increase research on ancient samples in Africa, in particular in the still poorly investigate regions of the Sahara.

## Materials and Methods

### Archaeological context and description of the samples

Takarkori is a large rockshelter in the Tadrart Acacus Mts., SW Libya (Fig. S1). It is located approximately 100 m above the wadi floor and has a well-preserved archaeological deposit. The excavations covered approximately 140 sq. m. The shelter was initially occupied by Late Acacus hunter-gatherers from ca. 10.2 to 8.0 ka. Although affected by severe erosional phenomena, the site preserves evidence of Early Pastoral Neolithic herders from approximately ca. 8.3 to 7.2 ca. The Middle Pastoral occupation is preserved and rich, with dates ranging between ca. 7.1 to 5.6 ka. Late Pastoral nomads seasonally frequented the site between ca. 5.9 and 4.4 ka.

During the excavations (four campaigns, between 2003 and 2006), we discovered a burial area in a recessed part of the shelter, very close to the rock wall. We unearthed the remains of 12 burials of different cultural phases, sex and age at death together with three further fragmentary remains from other areas of the shelter. Their state of preservation varies from badly fragmented parts of long bones to well-preserved and naturally mummified corpses, such as RS H1 and RS H9. Several papers describe the main features of the site^17,18,40–44^. Funerary practices, isotopic data on diet and mobility and cultural implications of Takarkori inhumations are discussed elsewhere^18^. Preliminary osteological and morphometric information are also available^38^.

Given the state of disturbance of the burial area, all inhumations have been directly radiocarbon dated, including TK RS H1 and H9 (Table S1).

Herein a synthesis of the main features of the two naturally mummified human remains object of the present molecular study.

#### TK RS H1

The skeletal remains of a 30–40 years old female were found in a shallow pit dug in organic sand. RS H1 shown anatomical parts naturally dried. The position of the body was contracted on the its left side (N-S oriented, looking at East). The skeleton was nearly complete: the right humerus, the right fibula and some of the thoracic vertebrae were missing. The disturbance to the burial is due to post-depositional processes, presumably linked to both the deposition of TK RS H9 and more recent disturbances. The only grave good found was a small figurine of a cow (?), made of local clay. The burial belongs to the very beginning of the Middle Pastoral phase (MP1).

#### TK RS H9

Immediately beneath TK RS H1 we found some partially dried anatomical districts of a skeletally mature individual (30–35), very likely a female. In particular, we excavated to complete and articulated pelvis, lying on its ventral surface, and portions of the lower limbs in anatomical connection. The legs were incomplete (complete femurs and portions of the two tibiae): the right leg was extended while the left one was strongly twisted beneath the pelvis. On the basis of the stratigraphic position, the remains of TK RS H9 were heavily affected by a post depositional disturbance. The burial is of Middle Pastoral phase (MP1).

### Sample preparation and sequencing

Several specimens were sampled from both bones and skin of the individuals TK RS H1 and TK RS H9 (Table S2) and analysed in the Molecular Anthropology Laboratory of the University of Florence, exclusively dedicated to ancient DNA analysis. Blanks as negative controls were used in all the experimental steps to monitor the absence of contaminants in reagents and environment. Bone and tooth samples were cleaned by removing the surface layer using a dentist drill with disposable tips and exposed under UV light (λ = 254 nm) for 45 minutes on each side. 100 mg of powder were sampled from inside the compact portion of the bones and from the dentine of the tooth root and used in DNA extraction. The skin fragments were washed with bidistilled DNA/RNA free water to eliminate residues of the soil, then exposed to UV lights and finally crumbled with a disposable scalpel. DNA was extracted from bone and tooth powder following a protocol designed for optimizing the retrieval of very short DNA fragments in highly degraded samples^45^; for skin samples QIAamp DNA Investigator Kit and EZ1 Qiagen robot were used. 30 µl of DNA extract were transformed into genetic library for each specimen following a double-stranded DNA protocol^46^ using a unique combination of two indexes per specimen. Negative controls were checked with both qPCR and Agilent 2100 Bioanalyzer DNA 1000 chip. After adapter ligation blanks had a concentration of 4–5 order lower than the biological samples, while indexing PCR products showed the presence of adapter-indexes dimers only. Libraries were then enriched for mitochondrial DNA following a multiplexed capture protocol^47^ and sequenced on an Illumina MiSeq run for 2 × 76 + 8 + 8 cycles.

### Sequence data processing and authenticity

After demultiplexing and sorting of the sequences according to the sample, raw sequence data were analysed using a pipeline specific for ancient DNA samples^48^. Adapters were clipped-off and paired-end reads with a minimum overlap of 10 bp merged in a single sequence using Clip&Merge version 1.7.4. Merged reads were then mapped on the revised Cambridge Reference Sequence, rCRS (GenBank Accession Number NC_012920) by CircularMapper in order to take into account the circularity of the mitochondrial genome. Duplicates were removed using DeDup, a tool that considers both ends of the fragments to recognize them as clonal. Reads with mapping quality below 30 were discarded. Results of mapping for each sample are shown in Table S3. A mean coverage of 72.38x was obtained for sample TK RS H1–1, 8.04x for sample TK RS H9-2 and 8.45 for sample TK RS H9-3, with more than 89% of the mitochondrion covered at least by 3 bases. For these reason, these three samples were processed for further analyses, while the other samples with lower coverage were discarded. Mapping reads for the three samples were analysed for deamination and length patterns using MapDamage 2.0^49^ and results are shown in Figs S2 and S3 respectively. The average fragment length is around 47 bp and presence of nucleotide misincorporation due to deamination increases at the ends of the reads resulting in an average frequency of 23% of CtoT at the first base at 5′ and GtoA at 3′ ends. These data highlight a considerable degradation of the genetic material compatible with the antiquity of the samples^8^ and the environmental conditions^10^. Schmutzi^50^, was used to estimate contamination levels and infer the final endogenous consensus. This approach estimates contamination levels based on deamination rates at the first bases on read termini, sequence length and a non-redundant database of human mitochondrial genomes. Estimates are shown in Table S4 and are compatible with absence of contamination. It also defines the endogenous consensus sequence, removing reads that possible belong to contaminants and mitigating the impact of deamination. Quality thresholds of 20, 30 and 40 were tested to obtain the final consensus sequence for the three samples. Consensus sequence was further checked by visual inspection of the mapping reads using the software Geneious v. R10.1^51^. Consensus sequences of samples TK RS H9-2 and TK RS H9-3 were first compared to each other as they belonged to the same anatomical element of the same individual. As expected, they showed to have the same haplotype, and it represents a further proof of the authenticity of the result confirmed by two independent experiments; for this reason the two samples were merged together in a single consensus representing the individual TK RS H9.

### Mitochondrial haplotype and haplogroup assignment

The sequences for the individuals TK RS H1 and TK RS H9 were uploaded to Haplofind^52^, Haplogrep^53^ and to Empop^54^ and aligned to each other and to the rCRS and the Reconstructed Sapiens Reference Sequence (RSRS)^55^ using ClustalX^56^ for a further visual assessment of the variants. Assignment by Haplofind, Haplogrep and Empop resulted in haplogroup N for both individuals with a score of 0.8 and 0.72 according to Haplofind and Haplogrep respectively. No perfect match was found with other sequences previously deposited in Empop or GenBank^57^. Polymorphisms as well as missing positions for the two samples according to rCRS and RSRS are shown in Table S5. They do not show derived alleles at positions 8701 and 16187. Interestingly, the ancestral state at position 8701 (which is a N-defining site for all modern lineages of the macro-haplogroup N observed to date) is shared with Oase 1^19^. This suggests that this lineage diverged from the stem of macrohaplogroup N. The two individuals TK RS H1 and TK RS H9 show the same haplotype with the only exception of some missing data in sample TK RS H9 that do not permit a complete comparison of all the sites of the mitochondrial genome. For this reason we cannot exclude that TK RS H9 retains some private mutations in these missing positions. Since the estimates of the mtDNA mutation rate are different^58^, we considered the higher rate available in literature in order to follow a conservative approach as indicated in^59^, resulting in 10 control region mutations in 327 generations according to genealogical data^60^. Considering the calibrated C14 dating ranges, a maximum distance of 879 and a minimum of 250 years separated the two individuals, corresponding to 35-10 generations of 25 years each. A maximum of 1.1 mutations for control region could have occurred in 36 generations between TK RS H1 and TK RS H9 if maternally related. Although some missing positions are present in TK RS H9 control region sequence that could mask possible mutations, all the positions considered to have fast or intermediate mutation rate^61^ are covered and present the same nucleotide of TK RS H1. Therefore, even if we cannot surely assess that the two individuals were related, the unicity of the haplotype and the sharing of both diagnostic and private mutations between the two sequences, indicate that the two individuals could be matrilineal relatives, as suggested also by the archaeological context.

### Phylogenetic analysis

We performed a Median Joining Network to better explore the phylogenetic position of the two ancient sequences. We selected 8 ancient and 536 modern sequences belonging to different sub-branches of the N haplogroup as well as to the haplogroup L3 and M, plus the rCRS and RSRS reference sequences (Table S6). Alignment was performed with the DNA Alignment software and then visually confirmed. Nucleotide position 16519 as well as the poly-C stretches and AC-indels at positions 16180–16193, 303–315, 515–524 and 573–576 were excluded from the phylogenetic analysis^62^. The Median Joining Network was calculated by Network v.5 (www.fluxus-engineering.com) giving equal weights to all nucleotide sites. The resulting network was drawn without applying pre- or post-processing steps and visualized by means of Network Publisher.

BEAST v1.8.0^63^ was used to produce a phylogenetic tree from a multi-sequence alignment containing the mtDNA sequence of TK RS H1, as representative of the haplotype found in Takarkori, and 209 published complete genomes of ancient and modern samples with lineage relevance for main haplogroups and sub-haplogroups (Tables S6 and S7). The best substitution model for the dataset, as determined according to Mega 7^64^, was the Generalised time-reversible with a fixed fraction of invariable sites and gamma distributed rates (GTR + G + I). In order to test the best models for the clock and the tree, a marginal likelihood estimation (MLE) using path sampling (PS) and stepping-stone sampling (SS) was performed for model comparison and best support assessment^65^ was used to test a strict clock and uncorrelated lognormal relaxed clock as clock models and a constant population size and a Bayesian skyline as tree models. The clock was calibrated using the other dated samples as calibration points^66^ (Table S7) by sampling with individual priors and setting an uniform distribution with the lower and upper limits of the date as confidence intervals. We further estimated the molecular age of TK RS H1. For each of the four different combinations of clock and tree models a MCMC run with 200,000,000 generations, sampling every 2,000, was performed. Effective sampling size (ESS) values and chain convergence were evaluated using Tracer v 1.6^63^. ESS values were higher than 200 for all the parameters in all the model combinations. The constant population size model, in combination with the strict clock rate, was overall best supported based on the MLE and chosen for the final analysis. The first 20% of iterations were discarded as burn-in and a Maximum Clade Credibility tree was obtained using TreeAnnotator v1.8.0^63^ and visualized with FigTree (http://tree.bio.ed.ac.uk/software/figtree/).

Sequence data are available for download through the National Center of Biotechnology Information as raw reads (PRJNA393833) and consensus sequences fasta files (Accession Numbers MF479727-MF479728).

## Supplementary information

Supplementary Materials

Supplementary Tables
