# Supplementary material for: Ancestral mitochondrial N lineage from the Neolithic ‘green’ Sahara
Source: Sci Rep. 2019 Mar 5;9:3530. doi: 10.1038/s41598-019-39802-1 (PMC6401177; doi:10.1038/s41598-019-39802-1)
Supplement: Supplementary file 1 — Supplementary Materials [file 41598_2019_39802_MOESM1_ESM.pdf]

Ancestral mitochondrial N lineage from the Neolithic ‘green’ Sahara

Stefania Vai[1], Stefania Sarno[2], Martina Lari[1], Donata Luiselli[2], Giorgio Manzi[3], Marina Gallinaro[4], Safaa Mataich[2], Alexander Hübner[5], Alessandra Modi[1], Elena Pilli[1], Mary Anne Tafuri[3], David Caramelli[1]*, Savino di Lernia[4,6]*

List of Supplementary Tables

Table S1. Radiocarbon dating for the two individuals subject to the molecular analysis.

Table S2. Sample information: for each sample genetically analyzed, laboratory ID and anatomical element are reported.

Table S3. Results for the mtDNA captures. mtDNA average coverage, average fragment length and misincorporation percentage at molecule termini are shown.

Table S4. Results of contamination estimates for the three libraries with higher mtDNA coverage.

Table S5. Polymorphisms and missing positions for the mtDNA of individuals TK RS H1 and TK RS H9 according to the Revised Cambridge Reference Sequence (rCRS) and the Reconstructed Sapiens Reference Sequence (RSRS).

Table S6. Dataset of ancient (in red) and modern (in black) sequences used for Network analysis. Modern samples with lineage relevance selected for the BEAST analysis are indicated with an asterisk (*).

Table S7. Dataset of ancient sequences used as tip calibration points in the Beast analysis.

List of Supplementary Figures

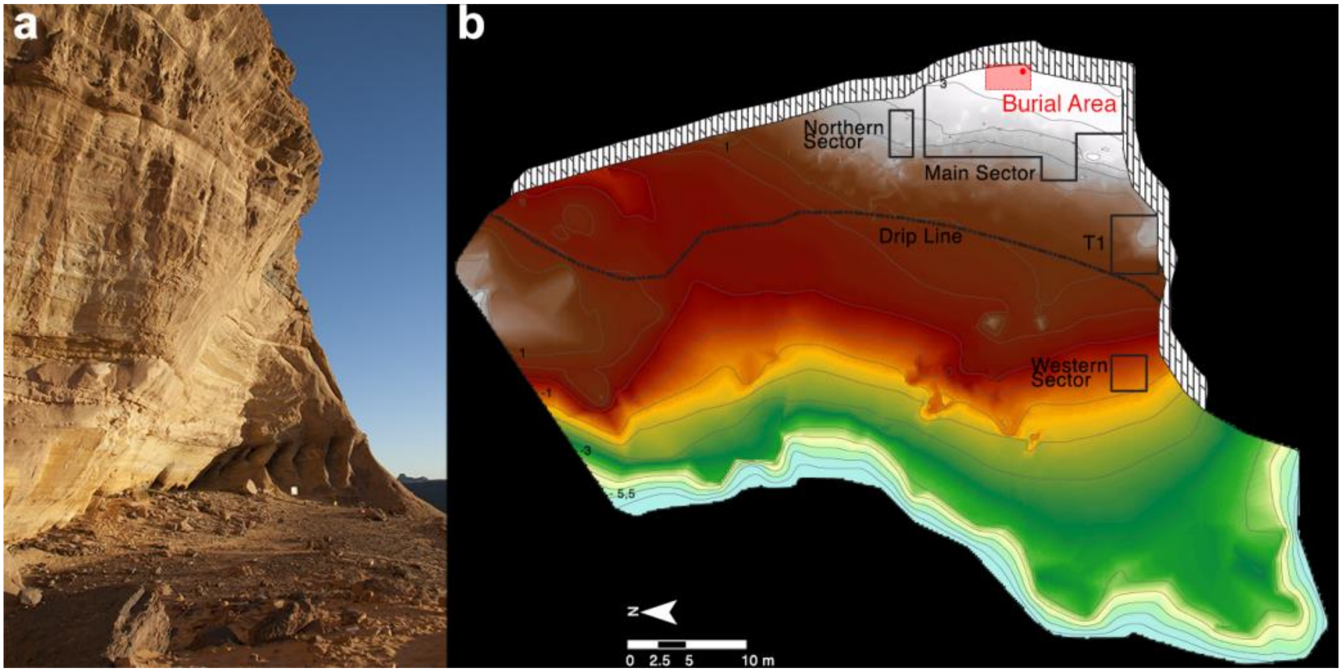

Figure S1. Takarkori Rockshelter seen from North (a) and Digital Terrain Model of the terrace, with location of the main sectors of excavation (b). In red the Burial Area and location of TK RS H1 and H9 (red dot).

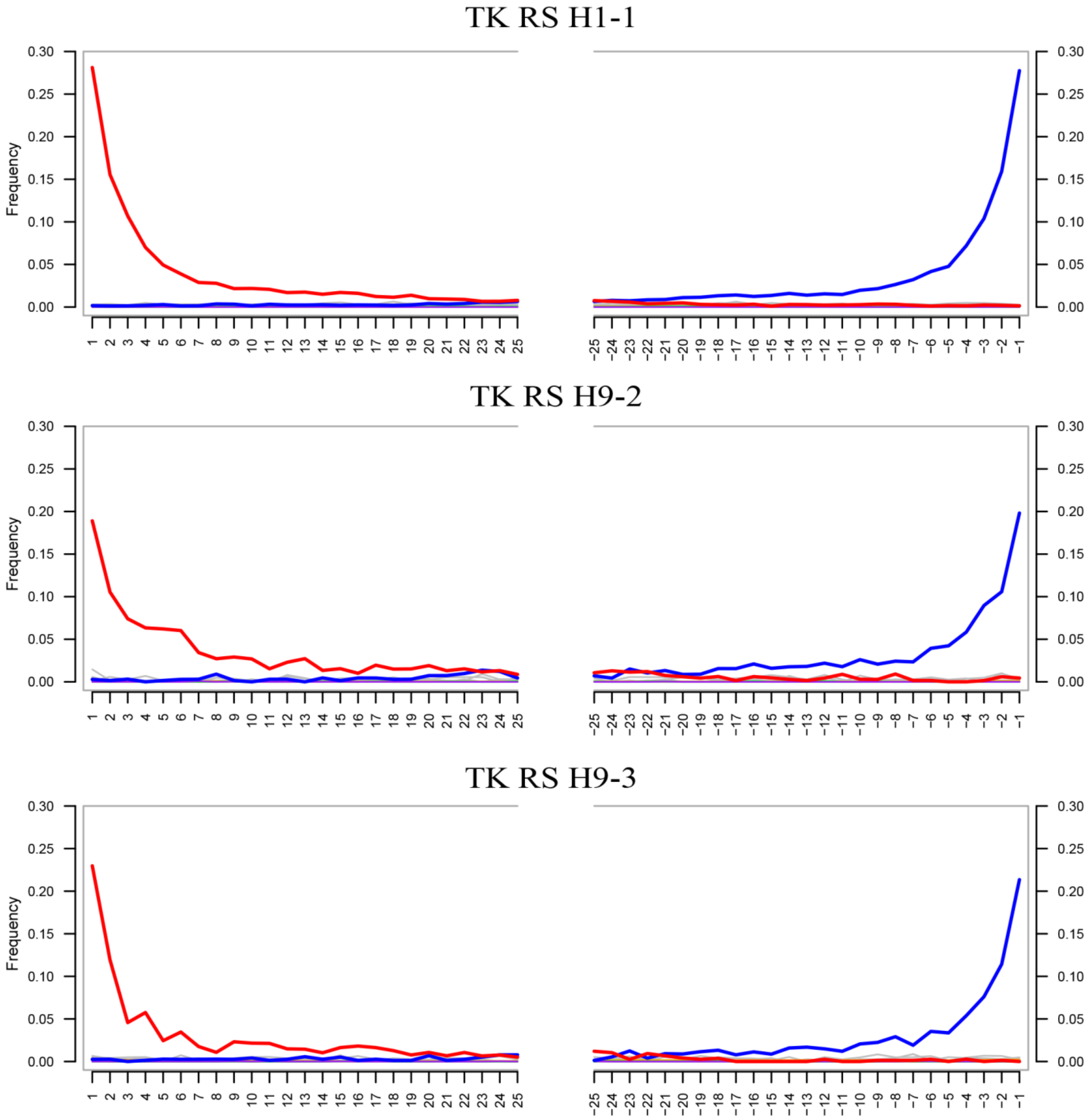

Figure S2. Misincorporation pattern for the sequences mapping on the reference for sample TK RS H1-1, TK RS H9-2 and TK RS H9-3. The CtoT deamination rate at the 5' end is shown with a red curve on the left, the GtoA deamination rate at 3' end is shown with a blue curve on the right.

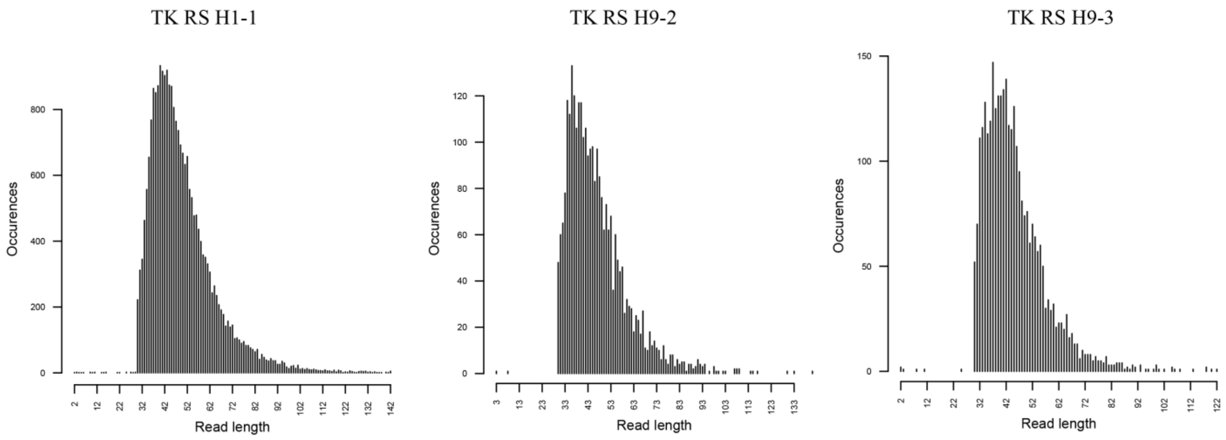

Figure S3. Fragment length distribution for the sequences mapping on the reference for sample TK RS H1-1 TK RS H9-2 and TK RS H9-3.

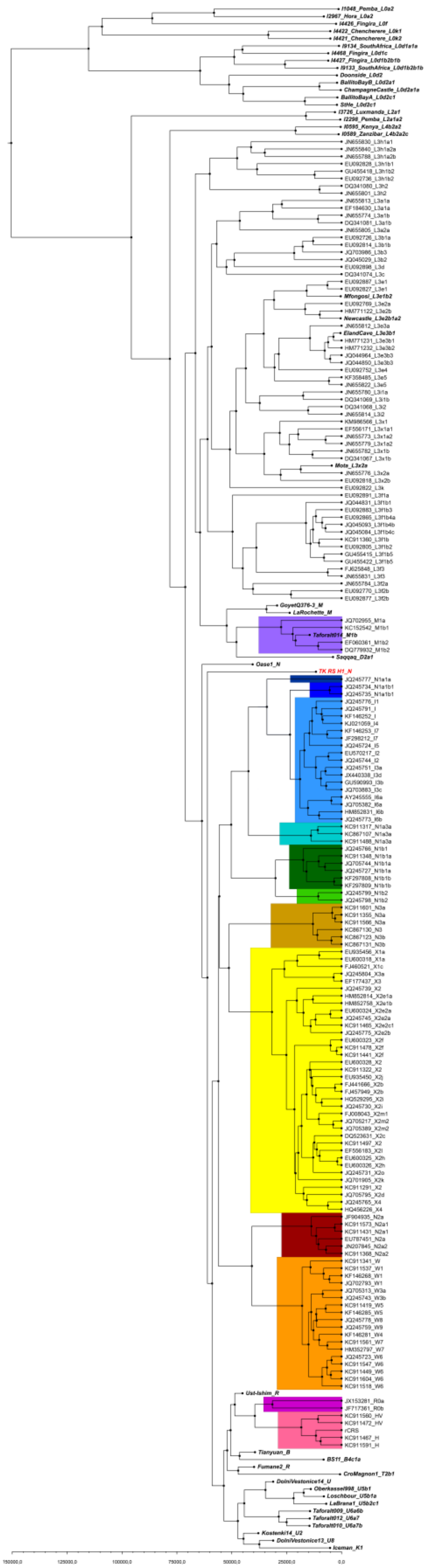

I1048_Pemba_L0a2
I2967_Hora_L0a2
I4426_Fingira_L0f
I4422_Chencherere_L0k1
I4421_Chencherere_L0k2
I9134_SouthAfrica_L0d1a1a
I4468_Fingira_L0d1c
I4427_Fingira_L0d1b2b1b
I9133_SouthAfrica_L0d1b2b1b
Doonside_L0d2
BallitoBayB_L0d2a1
ChampagneCastle_L0d2a1a
BallitoBayA_L0d2c1
StHe_L0d2c1
I3726_Luxmanda_L2a1
I2298_Pemba_L2a1a2
I0595_Kenya_L4b2a2
I0589_Zanzibar_L4b2a2c
JN655830_L3h1a1
JN655840_L3h1a2a
JN655788_L3h1a2b
EU092828_L3h1b1
GU455418_L3h1b2
EU092736_L3h1b2
DQ341080_L3h2
JN655801_L3h2
JN655813_L3a1a
EF184630_L3a1a
JN655774_L3a1b
DQ341081_L3a1b
JN655805_L3a2a
EU092726_L3b1a
EU092814_L3b1b
JQ703986_L3b3
JQ045029_L3b2
EU092898_L3d
DQ341074_L3c
EU092887_L3e1
EU092827_L3e1
Mfongosi_L3e1b2
EU092769_L3e2a
HM771122_L3e2b
Newcastle_L3e2b1a2
JN655812_L3e3a
ElandCave_L3e3b1
HM771231_L3e3b1
HM771232_L3e3b2
JQ044964_L3e3b3
JQ044850_L3e3b3
EU092752_L3e4
KF358485_L3e5
JN655822_L3e5
JN655780_L3i1a
DQ341069_L3i1b
DQ341068_L3i2
JN655814_L3i2
KM986566_L3x1
EF556171_L3x1a1
JN655773_L3x1a2
JN655779_L3x1a2
JN655782_L3x1b
DQ341067_L3x1b
Mota_L3x2a
JN655776_L3x2a
EU092818_L3x2b
EU092822_L3k
EU092891_L3f1a
JQ044831_L3f1b1
EU092883_L3f1b3
EU092865_L3f1b4a
JQ045093_L3f1b4b
JQ045084_L3f1b4c
KC911360_L3f1b
EU092805_L3f1b2
GU455415_L3f1b5
GU455422_L3f1b5
FJ625848_L3f3
JN655831_L3f3
JN655784_L3f2a
EU092770_L3f2b
EU092877_L3f2b
GoyetQ376-3_M
LaRochette_M
JQ702955_M1a
KC152542_M1b1
Taforalt014_M1b
EF060361_M1b2
DQ779932_M1b2
Saqqaq_D2a1
Oase1_N
TK RS H1_N
JQ245777_N1a1a
JQ245734_N1a1b1
JQ245735_N1a1b1
JQ245776_I1
JQ245791_I
KF146252_I
KJ021059_I4
KF146253_I7
JF298212_I7
JQ245724_I5
EU570217_I2
JQ245744_I2
JQ245751_I3a
JX440338_I3d
GU590993_I3b
JQ703883_I3c
AY245555_I6a
JQ705382_I6a
HM852831_I6b
JQ245773_I6b
KC911317_N1a3a
KC867107_N1a3a
KC911488_N1a3a
JQ245766_N1b1
KC911348_N1b1a
JQ705744_N1b1a
JQ245727_N1b1a
KF297808_N1b1b
KF297809_N1b1b
JQ245799_N1b2
JQ245798_N1b2
KC911601_N3a
KC911355_N3a
KC911566_N3a
KC867130_N3
KC867123_N3b
KC867131_N3b
EU935456_X1a
EU600318_X1a
FJ460521_X1c
JQ245804_X3a
EF177437_X3
JQ245739_X2
HM852814_X2e1a
HM852758_X2e1b
EU600324_X2e2a
JQ245745_X2e2a
KC911465_X2e2c1
JQ245775_X2e2b
EU600323_X2f
KC911478_X2f
KC911441_X2f
EU600328_X2
KC911322_X2
EU935450_X2j
FJ441666_X2b
FJ457949_X2b
HQ529295_X2i
JQ245730_X2i
FJ008043_X2m1
JQ705217_X2m2
JQ705389_X2m2
DQ523631_X2c
KC911497_X2
EF556183_X2l
EU600325_X2h
EU600326_X2h
JQ245731_X2o
JQ701905_X2k
KC911291_X2
JQ705795_X2d
JQ245765_X4
HQ456226_X4
JF904935_N2a
KC911573_N2a1
KC911431_N2a1
EU787451_N2a
JN207845_N2a2
KC911368_N2a2
KC911341_W
KC911537_W1
KF146268_W1
JQ702793_W1
JQ705313_W3a
JQ245743_W3b
KC911419_W5
KF146285_W5
JQ245778_W8
JQ245759_W9
KF146281_W4
KC911561_W7
HM352797_W7
JQ245723_W6
KC911547_W6
KC911449_W6
KC911604_W6
KC911518_W6
Ust-Ishim_R
JX153281_R0a
JF717361_R0b
KC911560_HV
KC911472_HV
rCRS
KC911467_H
KC911591_H
Tianyuan_B
BS11_B4c1a
Fumane2_R
CroMagnon1_T2b1
DolniVestonice14_U
Oberkassel998_U5b1
Loschbour_U5b1a
LaBrana1_U5b2c1
Taforalt009_U6a6b
Taforalt012_U6a7
Taforalt010_U6a7b
Kostenki14_U2
DolniVestonice13_U8
Iceman_K1
150000,0
125000,0
100000,0
75000,0
50000,0
25000,0
0,0

Figure S4. Bayesian phylogenetic tree constructed for the Takarkori sample TK RS H1 and 209 published complete genomes of ancient and modern samples. All branches are shown in detail. The major mitochondrial lineages are indicated in different colours as in the Median Joining Network (Fig. 2).
